# Pupillometry in Chinese Female Patients with Depression: A Pilot Study

**DOI:** 10.3390/ijerph110202236

**Published:** 2014-02-21

**Authors:** Jikun Wang, Yaodong Fan, Xudong Zhao, Nanhui Chen

**Affiliations:** 1 Key Laboratory of Brain Functional Genomics, MOE & STCSM, School of Psychology and Cognitive Science, East China Normal University, Shanghai 200062, China; 2 Department of Psychosomatic Medicine, Shanghai East Hospital, Tongji University School of Medicine, Shanghai 200092, China; E-Mail: zhaoxd62@gmail.com; 3 Department of Neurosurgery, The Third Affiliated Hospital of Kunming Medical College, Kunming 650032, China; E-Mail: 136090365@qq.com; 4 Kunming Institute of Zoology, Chinese Academy of Sciences, Kunming 650223, China

**Keywords:** depression, pupillary light reflex, pupillometry, Chinese females

## Abstract

The pupillary responses to light in patients with depression and normal controls were evaluated among Chinese females. Four parameters related to the pupil were assessed using a video-based pupillometer. The results showed that there were significant differences in the pupil area in the darkness and the pupil area at the peak of constriction between depressed patients and normal controls.

## 1. Introduction

Depression is a common mental disorder featuring as the most prominent symptom a low mood. Significant deficits in neurocognitive function such as poor attention and impaired memory processing due to dysfunction of the central cholinergic system, also accompany depression [1,2]. Pupillometry has been applied as an easy non-invasive method to assess the central cholinergic system function [3]. The pupillary constriction and the re-dilation process are governed by the action of the Edinger–Westphal nucleus (EW) and the Locus Coeruleus (LC) through their projections to the ciliary ganglion and the ciliospinal center of Budge–Waller, respectively [3]. Acetylcholine (ACH) is the primary neurotransmitter that mediates the pupillary light reflex. In addition, the EW nucleus is cholinergic in nature [4]. Therefore, the pupillary light reflex has been investigated in disorders with significant cholinergic impairment, such as Parkinson's disease [4], Alzheimer's disease [5], depression [6], and generalized anxiety disorder [7].

The evidence concerning pupillary light response in depressed patients is complex. For example, Sokolski and DeMet [8] hypothesized that muscarinic receptor sensitivity could increase during depression, and their data suggested that depression possesses both peripheral and central muscarinic hypersensitivity [9,10]. This hypersensitive pupil reaction may be trait-dependent and regulated by M3 muscarinic receptors through a pathway that involves G-protein-mediated activation of phosphatidylinositol.

Sokolski and DeMet also proposed that increased cholinergic sensitivity in depressed patients results in pupillary constriction [11]. In addition, they [12] further reported that younger depressed patients exhibited larger pupil areas than age-matched normal controls, which could reflect an increased demand on the noradrenergic system. The maximal pupillary dilation in depressed patients has been shown to decrease with aging [12], which may result from the loss of cortical noradrenergic neurons. Fountoulakis *et al.* [6] reported that there was a marginally significant decrease in the latency to constriction in patients with depression. Siegle *et al.* [13] used pupillary analysis as an index to assess the effect of cognitive therapy on depression for several reasons: (1) pupillary responses reflect cognitive and emotional processing; and (2) the pupillary reaction reflects relevant brain mechanisms of limbic activity and cognitive control [14]. Research by Fountoulakis *et al.* [6] suggested that cholinergic and other neurotransmitters may play a significant role in development and the modulation of emotion.

Although several studies have applied pupillometry analysis in depressed patients of European origin, there is a dearth of such analysis for depressed patients in the Chinese population. In addition, pupillary analysis technology has improved substantially and our knowledge of depression has advanced over time too, making it necessary to re-evaluate the pupillary light reflex in depressed patients. Furthermore, there is a need for pupillary analysis in depressed patients in other ethnic groups because depressive symptoms may vary among patients with different genetic background. Thus, the aim of the present study was to evaluate the pupillary light reflex in depressed patients and normal controls in China, especially for female Chinese individuals aged 21 to 39.

## 2. Methods

### 2.1. Participants

Patients with first-episode depression were recruited from the outpatient clinic of the Psychiatry Department at the Affiliated East Hospital of Tongji University in Shanghai, China. Normal controls were recruited by community advertisements. Patients were interviewed with the Structured Clinical Interview for DSM-IV (SCID) [15]. The patient group consisted of 15 depressed female patients with a mean age of 31.1 years (SD = 3.5, range = 23–38), and diagnosed with major depressive disorder based on DSM-IV criteria [16].The control group consisted of 15 female controls with a mean age of 31.9 years (SD = 4.7, range = 22–38). The normal controls were free of medications for at least two weeks prior to the pupillary light reflex tests. The study was approved by the IRB of Tongji University School of Medicine, and written consent was obtained from each participant after the study was explained in detail.

### 2.2. Pupillary Analysis

Pupillary dilation was assessed during each participant’s first visit to the hospital. The pupil dilation from the left eye was assessed in the morning from 8:00 a.m. to 11:00 a.m. in a moderately lit room. A manual monocular pupillometer (Kunming Yilikete Co., Kunming, China) was used to video-record the pupillary light reflex [the resolution was 320 × 240 pixels, and the frame rate was 15 frames-per-second using the gray-level technique (8 bit)]. Before testing, the participant was asked to close her eyes and took a rest for 10 min. During the testing, the participants held the pupillometer which had eye covers to protect the eyes from light. While holding the pupillometer, the participant was asked to open her eyes for 2 min to adapt to the darkness. The participant was then asked to look straight ahead, and a red light was shone on the participant’s right eye inducing the shrinkage of both pupils. The red light was used to stimulate the alertness of depressed patients [17,18]. A uniform wavelength infrared light was focused on the left eye using the pupillometer. The indirect light reflex video of the left pupil was recorded by a camera in the pupillometer for further analysis. A computer software calculated the area of the pupil for each frame of the video to yield a pupil area curve for the pupillary light reflex.

The pupil recognition algorithm was based on a circular Hough transformation [19]. The video data were analyzed by Matlab software (MathWorks, Natick, MA, USA), and the Matlab code was customized by our laboratory. Blinks were eliminated by local regression, and the data were further smoothed using the median filter.

Three trials were performed for each participant, and each trial consisted of three stages: the first stage was 1 s before dark adaptation ended, the second stage was when the light in the pupillometer was turned on for 2 s and the third stage was when the light was turned off. The participant was asked to hold the pupillometer for at least 30 s until the pupil re-dilation to its maximum size was measured.

### 2.3. Data Analysis

Independent t tests were used to compare the data between the depressed patients and the normal controls. Four pupillary parameters were calculated, including the pupil area in the darkness (in mm^2^), the pupil area at the peak of constriction (in mm^2^), the latency time for constriction (in s) and the duration of pupillary constriction (in s). The parameters of pupillary light reflex included the pupil area in darkness (which means the pupil area when they are in darkness), pupil area in largest constriction (which means the pupil area when constricting the most), latency for constriction (which means the time spent from the beginning of light to the constriction of pupils), and the duration of constriction (which means the time spent from the beginning of pupillary constriction to the largest constriction of the pupil).

## 3. Results

The results showed that the pupil area in the darkness and the pupil area at the peak of constriction were larger in depressed patients compared with normal controls and there were significant differences in the pupil area in darkness and the pupil area at the peak of constriction between the depressed patients and the controls (Table 1). There was no significant difference, however, in the latency time to constriction or the duration of constriction between the two groups.

**Table 1:** Mean and standard deviation of pupillary variables in depressed patients and normal controls.

Items	Depressed Patients (*n* = 15)		Normal Controls (*n* = 15)		*p*
	Mean	SD	Mean	SD	
Age	31.1	3.5	31.9	4.7	0.474
Pupil area in darkness	32.38	5.40	26.00	5.12	0.002 **
Pupil area in largest constriction	20.77	5.79	16.86	3.84	0.039 *
Latency for constriction	0.29	0.06	0.27	0.04	0.213
Duration of constriction	2.11	0.16	2.03	0.22	0.287

*p*-Values reflect the results of t-test between these two diagnostic groups. * *p* < 0.05, ** *p* < 0.01.

## 4. Discussion

The present study used a modern optical method to assess the pupil responses to light in first-episode depressed patients. To the best of our knowledge, this is the first study to compare pupil dilation in response to light between depressed patients and normal controls in the Chinese population. The pupil area in the darkness before light stimulation was greater in the depressed patients than in the normal controls. This finding is consistent with a previous study in which younger patients with depression exhibited a larger pupil area than age-matched normal controls [12]. This finding could reflect an increased demand for the noradrenergic system, and in support of this, another study has suggested that depression is associated with lower levels of noradrenaline (NOR) [2].

A previous study has shown that the largest amplitude of pupil constriction may be a measure of ACH activity because NOR activity is already offset [6]. Our results show that the pupil area at the peak of constriction was greater in the depressed patients compared with the normal controls, supporting the hypothesis that ACH activity decreases in depression; however, Fountoulakis *et al.* [6] did not find any significant difference in ACH activity in depressed patients compared with controls.

Changes in pupil during constriction reflect cholinergic effects [20]. The latency to constriction is a marker of the balance between ACH and NOR, since the constriction of the pupil begins when ACH activity plus NOR blockade by ACH, overcome NOR activity [6]. Our results showed that the latency to constriction was significantly longer in depressed patients than in normal controls. Fountoulakis *et al.* [6] explored the pupillary responses to light and found a significant difference in the latency to constriction between depressed patients and normal controls suggesting that the activity of both ACH and NOR is decreased in depression. Interestingly, the reduction in NOR activity was greater than that in ACH activity. It is noteworthy that the present results focused on dynamic pupillary response rather than static pupil size control, suggesting the existence of lower level of NOR and ACH hypoactivity in depressed patients.

Other studies have also investigated the pupillary response in depressed patients. For example, Sokolski and DeMet showed that depressed patients exhibited increased cholinergic sensitivity resulting in pupillary constriction [8,11]. Interestingly, one study did not find a significant difference in pupillary light reflex between depressed patients and normal controls [9]. In addition, another study demonstrated that the maximum velocity and the maximum acceleration of re-dilation of the pupils were decreased in depressed patients [21], supporting the monoamine hypothesis of depression and suggesting that norepinephrine is the major neurotransmitter related to the re-dilation of the pupil.

There are a number of hypotheses on the development of depression. For example, the adrenergic-cholinergic balance hypothesis proposed that depression involves a hypercholinergic state [22], based on studies showing that depressed patients exhibited heightened cholinergic reactions to pilocarpine eyedrops [8]. In contrast, patients with more severe mania have been shown to exhibit decreased cholinergic tone [12]. Taken together, these studies suggest that mood is associated with the degree of cholinergic tone, and cholinergic dysfunction may be manifested in the peripheral and the central nervous systems. Anticholinergic drugs have been demonstrated to be a potential treatment for depression; however, the effects of the treatment have yielded conflicting results [23,24]. Hence, cholinergic hypersensitivity has been considered as a personality trait that predisposes individuals to depression-like sensitivity to stress rather than depression itself [25]. In summary, cholinergic deficits may explain the cognitive symptoms of depression (e.g., poor attention and concentration and impaired memory and information processing) rather than the affective symptoms [2].

The monoamine hypothesis of depression proposed that depression is associated with lower levels of brain monoamines, such as serotonin, NOR and dopamine [2], and mounting evidence has supported this hypothesis. For example, the mechanisms of action of many classes of antidepressant drugs involve elevating monoamine levels [26].

The understanding of depression has markedly increased over time. Today, major depressive disorder is thought to be a multifactorial syndrome that consists of disruptions in mood, cognition and other processes including sleep, appetite and libido [27]. Because depression is a complex mental disorder, it is unlikely that the neuronal system alone can fully explain the nature of depression [2]. For example, the loss of pleasure may result from dopaminergic and noradrenergic impairments [28], and the cognitive symptoms associated with depression could be explained by cholinergic dysfunction [2]. Interestingly, previous studies have shown that subjects with depression exhibit a variety of symptoms, and the influence of multiple neurotransmitters on the pathogenesis of depression may explain why previous studies on pupil analysis in depression have reported contradictory results. Further studies are needed to replicate the results that the pupil area in the darkness and the pupil area at the peak of constriction were larger in the depressed patients compared with the normal controls and to discriminate the depressed patients with different symptoms (e.g., delineate between patients with and without cognitive symptoms).

The present results showed that the pupil area in the darkness and the pupil area at the peak of constriction were greater in the depressed patients than the normal controls, suggesting that pupil analysis could be an easy noninvasive method to discriminate depressed patients from normal controls. Other studies have also proposed that pupillary responses may provide a simple noninvasive method to evaluate cholinergic sensitivity in patients with affective disorders [29].

There are several limitations intrinsically associated with the present study. First, larger patient groupa including male patients are necessary to further substantiate the results. Secondly, it is necessary for further studies to include more pupillary variables. Thirdly, since 2 min is too short for dark-adaptation resulting in pupil size alteration during the test, a longer adaptation time is needed in future studies to optimize the test paradigm. Although it appears that we uncovered an alteration of pupil size in depressed patients, mechanistic studies on possible causes of this effect are needed. Topical blocking agents such as dapiprazole could be applied to determine whether or not this is an enhancement of the peripheral adrenergic pathway. Finally, the pupillary light reflex could not be simplified into simply cholinergic and adrenergic components since the light reflex starts with a complicated process of phototransduction in the retina, proceeding to the pretectal area where an unknown neurotransmitter activates an interneuron that projects to the EW nucleus. This nucleus is under inhibitory control and in turn alters the size of the pupil and the extent of the light reflex. Thus, further studies are necessary to investigate the involvement of cholinergic or noradrenergic system in this process. In summary, pupillometry could be an easy noninvasive method that exhibits a broad range of clinical implications. In addition, pupillometry may be useful for the investigation of early cholinergic impairment, such as the cognitive symptoms observed in depression.

## 5. Conclusions

The findings suggest that the pupil analysis could be an easy noninvasive method to discriminate depressed patients from normal controls, and pupillary responses may provide a simple noninvasive method to evaluate cholinergic sensitivity in patients with affective disorders.
